# Characterization of Dengue Virus Type 2: New Insights on the 2010 Brazilian Epidemic

**DOI:** 10.1371/journal.pone.0011811

**Published:** 2010-07-28

**Authors:** Camila Malta Romano, Andréia Manso de Matos, Evaldo Stanislau A. Araújo, Lucy Santos Villas-Boas, Wanessa Cardoso da Silva, Olímpia M. N. P. F. Oliveira, Karina I. Carvalho, Ana Carolina Mamana de Souza, Celia L. Rodrigues, José Eduardo Levi, Esper G. Kallas, Claudio Sergio Pannuti

**Affiliations:** 1 Departamento de Moléstias Infecciosas e Parasitárias – (LIMHC), Instituto de Medicina Tropical de São Paulo e Faculdade de Medicina, Universidade de São Paulo, São Paulo, Brazil; 2 Disciplina de Imunologia Clínica e Alergia (LIM-60), Faculdade de Medicina, Universidade de São Paulo, São Paulo, Brazil; 3 Hospital Ana Costa, Santos, Brazil; BMSI-A*STAR, Singapore

## Abstract

Dengue viruses (DENV) serotypes 1, 2, and 3 have been causing yearly outbreaks in Brazil. In this study, we report the re-introduction of DENV2 in the coast of São Paulo State. Partial envelope viral genes were sequenced from eighteen patients with dengue fever during the 2010 epidemic. Phylogenetic analysis showed this strain belongs to the American/Asian genotype and was closely related to the virus that circulated in Rio de Janeiro in 2007 and 2008. The phylogeny also showed no clustering by clinical presentation, suggesting that the disease severity could not be explained by distinct variants or genotypes. The time of the most recent common ancestor of American/Asian genotype and the São Paulo and Rio de Janeiro (SP/RJ) monophyletic cluster was estimated to be around 40 and 10 years, respectively. Since this virus was first identified in Brazil in 2007, we suggest that it was already circulating in the country before causing the first documented outbreak. This is the first description of the 2010 outbreak in the State of São Paulo, Brazil, and should contribute to efforts to control and monitor the spread of DENVs in endemic areas.

## Introduction

Dengue is the most prevalent arthropod-born viral disease in tropical and subtropical regions, infecting 50 to 100 million people annually [1]. Cases in Central and Latin America have increased almost five-fold in the last 30 years. In 2008, up to one million cases were reported in the Americas, with higher number of deaths occurred in the Southern Cone [2]. In the last two decades, Brazil has been responsible for more than 60% of the total reported dengue fever cases in the Americas [2]. The continuing occurrence in resource limited countries and the lack of novel therapeutic approaches or an effective vaccine have made dengue fever to be considered a neglected disease. Dengue surveillance is deprived in most countries and no existing model for predicting an outbreak in endemic regions is available.

The mechanisms by which dengue virus (DENV) causes severe illness remain to be elucidated. Both viral and immune host factors seem to contribute to the level of the pathogenicity [3], [4], [5], [6]. Immunity induced by natural infection is believed to provide serotype-specific lifelong protection. On the other hand, previous infection by a distinct serotype increases the risk for dengue hemorrhagic fever and dengue shock syndrome (DHF/DSS) [5], [7].

In the Americas, DHF was first reported in Cuba in 1981 after the introduction of the DENV2 genotype from Southeast Asian (SEA) [8], [9]. Since then, other countries in the Americas have reported DHF associated with such DENV2 genotype, but not with the American genotype [9]. In Brazil, DENV2 was first identified in the State of Rio de Janeiro in 1990, where the first cases of DHF and DSS were documented [10]. It was followed by a fast spread of DENV2 across the country [11], [12], becoming endemic in some areas [13] and causing severe clinical forms [14]. Genomic analysis performed on DENV2 Brazilian isolates identified the co-circulation of two distinct lineages of the American and Asian genotypes [15], [16]. In a recent study, Oliveira *et al*. reported that the virus circulating in the State of Rio de Janeiro in 2007 and 2008 was genetically distinct from 1990 and 1998 isolates from respective epidemics, suggesting a local evolution of the DENV2 strain or, alternatively, a co-circulation of distinct lineages in the country [15]. In the beginning of 2010, several dengue outbreaks have been documented in many regions of Brazil, with the co-circulation of three serotypes (DENV1, 2, and 3). By the end of March 2010, the São Paulo State Healthy Department reported more than 34,000 cases of dengue, more than 800 only in the coast cities of Guarujá and Santos, where severe clinical presentations have been observed (64 deaths related to dengue occurred in the same time frame in the São Paulo State) [17].

In this study we characterized at molecular and phylogenetic levels the DENV2 strain causing the current epidemic in the coastal cities of Guarujá and Santos and surrounding areas. Using coalescence approaches, we also estimated the time (in years) that this specific lineage entered in Brazil.

## Methods

### Ethics Statement

The current project was conducted after *Hospital das Clínicas* - University of São Paulo's Institutional Review Board (CAPPesq) approval, under protocol #0652/09. Written informed consent was obtained from all participants.

### Clinical samples and virus Isolation

Serum samples were obtained from patients with clinical suspected dengue fever at the *Ana Costa* Hospital located in the city of Santos, State of São Paulo. DENV2 was successfully isolated from 18 serum samples by inoculation into monolayers of C6/36 *Aedes albopictus* cell line (ATCC number CRL-1660). Supernatants from cell cultures presenting cytopathic effect compatible with DENV infection were assayed by reverse transcriptase polymerase chain reaction (RT-PCR) to amplify serotype-specific viral genome.

### RNA extraction and RT-PCR

Viral RNA was isolated directly from 140 µl of cell culture supernatants using the QIAamp Viral RNA Mini Kit (Qiagen, Valencia, USA) following the manufacturer's instructions. cDNA was synthesized using the High Capacity cDNA Reverse Transcription Kit (Applied Biosystems, Foster City, CA). A total reaction volume of 20 µl was made with 10 µl of RNA and 10 µl of mix, containing 2.0 µl 10× buffer, 0.8 µl dNTP Mix (100 mM), 2.0 µl 10× random hexamer primers, 1.0 µl reverse transcriptase and 4.2 µl nuclease-free water.

### PCR amplification and sequencing

A 665 bp of envelope region corresponding to nucleotides 1857 to 2522 of DENV2 complete genome (GenBank reference sequence DQ181801) was amplified by PCR using following primers specific for DENV2: D2_EnvF -5′- AARRTYGTRAARGAAATAGCAGAA-3′ and D2_EnvOutR- 5′-CTGTCCAYGTRTGYACGTTRTCT-3′. The thermal profile for amplification was 38 cycles of 45 seconds of denaturation at 94°C, 1 minute of annealing at 56°C, and 1 minute of extension at 72°C. The PCR products were purified for elimination of primer dimmer and excess oligonucleotides with the PureLink Quick Gel extraction Kit (Invitrogen), with few adaptations for direct purification of PCR products in solution. Sequencing reactions were performed with BigDye Terminator kit v.3.0 (Applied Biosystems, Bedford, MA) and one of each primer used in sequencing reaction. The eletropherograms were analyzed and consensus sequences were achieved with CodonCode Aligner v.3.0 (available at http://www.codoncode.com/).

### Phylogenetic and coalescent analysis

The 18 sequences generated were aligned together with reference sequences downloaded from GenBank using the ClustalX program [18]. An initial dataset including our sequences and reference sequences from all four serotypes (n = 93) was used to confirm that our samples were truly DENV2. A second dataset included our samples and reference sequences of all DENV2 genotypes known to cause outbreaks around the word (n = 91). The phylogenetic trees were reconstructed using a Bayesian Markov Chain Monte Carlo (MCMC) framework implemented in BEAST v1.5.3 [19]. The GTR+G substitution model was used as the best model obtained in MODELTEST [20]. Also, the previously estimated substitution rate for DENV2 of 6.5×10^−4^ s/s/y [21] was used to obtain the time of the most recent common ancestor (TMRCA) for the American/Asian genotype and also for the Brazilian DENV2. The Bayesian Skyline plot (BSL) was performed under strict and relaxed uncorrelated lognormal molecular clock. The molecular clock that best fitted the data was chosen by Bayes factor (BF) comparison. Convergence of parameters during the MCMC was inspected with Tracer v.1.4 [19], with uncertainties addressed as 95% highest probability density (HPD) intervals. In all cases, ten million chains were sufficient to achieve the convergence of all parameters (ESS>200). The trees were sampled at each 1000 steps, resulting in a final file of 10,000 trees. These trees were summarized in a maximum clade credibility (MCC) tree using TreeAnotator (part of the BEAST package), and visualized in FigTree v.1.2.2 (http://beast.bio.ed.ac.uk/FigTree).

## Results

**Figure 1:**
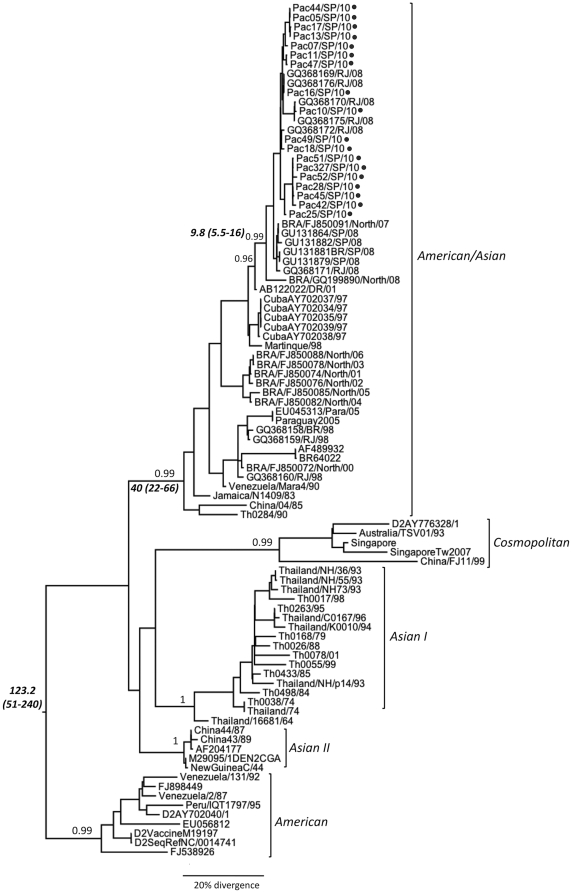
Phylogenetic tree of DENV2 Brazilian samples. The maximum clade credibility (MCC) tree was inferred for 18 partial envelope sequences from the city of Santos, State of São Paulo and DENV2 reference sequences. The posterior probabilities of the key nodes are depicted above the respective node. The time of the most recent common ancestor (TMRCA) of the more recent lineage, American/Asian genotype and serotype 2, with upper and lower intervals in parenthesis, are depicted in bold in the respective nodes. Black full circles identify the sequences generated in this study. Brazilian sequences are identified according to the region of origin: “North“ (from Northeast region) and “SP” or “RJ” (from Southeast region). When available, the sampling year was described in the end of the sequences.

An initial phylogenetic tree including all DENV serotypes confirmed, with high posterior probability, that our samples belong to serotype 2 (supplementary Figure S1). The maximum clade credibility (MCC) tree (Figure 1) showed that the DENV2 isolates circulating in 2010 in Guarujá and Santos clustered within the American/Asian genotype and were closely related to viruses that caused the epidemics in Rio de Janeiro during 2007 and 2008 [15]. Although this study included only two patients with severe dengue (patients 28 and 42, Table 1), the phylogenetic tree showed no segregation by clinical manifestations, since mild and severe dengue fever cases samples clustered together. Moreover, we found no differences at aminoacid level in the envelope region among patients with distinct clinical presentations. However, it must be taken into account that differences in clinical manifestation may not be evident by our analysis, since they included a limited number of patients and encompassed only partial envelope gene. Age, gender and further clinical data are described in Table 1.

**Table 1:** Characteristics of the 18 patients sampled in Santos (2010) with DENV2 disease.

Patient ID	City	Gender	Age (years)	Platelets (×1000/µl)	Clinical presentation*	Death
05	São Vicente	Female	25	174	Dengue fever	No
07	Santos	Female	34	253	Dengue fever	No
10	Guarujá	Female	37	152	Dengue fever	No
11	Santos	Male	53	179	Dengue fever	No
13	São Vicente	Female	74	120	Dengue fever	No
16	Santos	Female	22	200	Dengue fever	No
17	Santos	Male	47	237	Dengue fever	No
18	ND**	Female	16	39	ND**	Yes
25	ND**	Male	50	48	Dengue fever	No
28	Santos	Male	79	17	Severe (DSS)	Yes
42	Santos	Female	86	20	Severe (DHF)	No
44	São Vicente	Female	29	216	Dengue fever	No
45	Santos	Male	31	230	Dengue fever	No
47	Santos	Female	31	147	Dengue fever	No
49	Santos	Male	51	158	Dengue fever	No
51	Santos	Female	53	135	Dengue fever	No
52	Santos	Female	46	132	Dengue fever	No
327	Santos	Female	0***	ND**	Dengue fever	No

*The patients presenting severe dengue must present at least one of the follow clinical findings: plasma leakage (leading to DSS and/or respiratory distress); severe bleeding and severe organ involvement. This has been defined by the World Health Organization Guideline, 2009.

**ND not determined.

***one week.

Importantly, the monophyletic cluster of the recent Brazilian DENV2 was closely related to viral strains from Cuba and the Dominican Republic, with small genetic distance and high posterior probability, suggesting that this lineage was imported from the Caribbean.

The coalescent analysis based on the dataset containing the five DENV2 genotypes revealed that the lineage causing the epidemics in the last four years in Brazil was introduced in the country at least at 5.5 years ago (median value of 9.8 years ago, and upper value of 16 years ago). Importantly, Figure 1 also showed that our estimates for the time of the most recent common ancestor (TMRCA) of the key nodes covered essentially the same range as those estimated by previous reports [21], [22], further supporting our results. The TMRCA data described here were obtained under relaxed uncorrelated molecular clock. Although the relaxed clock was the best model by Bayes Factor comparison, the coalescent dates obtained under the strict clock were similar (Supplementary Files S1 A and B).

The sequences generated in this work were submitted to GenBank database under accession numbers HM185186 to HM185202.

## Discussion

Our phylogenetic analysis based on the partial DENV2 envelope gene sequencing showed that viruses causing the current epidemic in the cities of Guarujá and Santos, as well as surrounding areas, were sister taxa of those sampled two years ago in the cities of Rio de Janeiro and Ribeirão Preto. The presence of *Aedes aegypti* and the frequent traveling amongst such regions are the necessary ingredients for viral spread through viremic patients moving between these cities.

It was interesting to find that the lineage causing outbreaks in Brazil in the end of 90's, the isolates from Northeast Brazil between 2001 and 2006, and the viruses representing more recent epidemics (2007 to 2010) in São Paulo and Rio de Janeiro States segregated into temporal sub-clades (Figure 1). Several longitudinal studies have also observed temporal clustering of dengue, as well as marked patterns of geographical structure [23], [24], [25]. The circulation of distinct lineages of DENV2 in Brazil had already been observed before [15] and was ascribed either to independent introductions in different time points or as a result of local evolution.

The sequences distribution in the phylogenetic tree indicated that the oldest Brazilian DENV2 are more related to viruses from South American countries (Paraguay and Venezuela), and the more recent isolate appears to be a result of a re-introduction from the Caribbean (Martinique, Cuba, and the Dominican Republic). In fact, migration of DENV2 from the Caribbean islands into America's mainland was reported to be relatively frequent [26]. However, due to the low availability of sequences from other South American countries, we could not precisely determine the location of such introduction.

It is well known that DENV is characterized by frequent clade replacements (*i.e.*, the replacement of the successful lineage present in one sampling period to the next) [25]. Also, this “lineage turnover” phenomenon appears to be a consequence of the stochastic forces and host immunity pressures, which ultimately determines the prevalence of a specific clade or serotype in a particular season [25], [27]. This model agrees to both conditions observed during the recent epidemics in Brazil: (*i*) the shift from DENV3 to DENV2 prevalence in the Southern region and, (*ii*) the emergence of a genetically distinct lineage of DENV2 in 2007, possibly caused by the re-introduction of viruses imported from the Caribbean. Although phylogenetic data show that some strains can persist and locally evolve, our data on DENV2 in Brazil suggest that the re-introduction may be important for the dynamics of the epidemics.

The evolutionary history of dengue virus is recent and DENV2 is estimated to have emerged between 120 and 215 years ago [21], [22], [28], which is in line with our prediction of 123 years for this serotype. Additionally, we estimated that the American/Asian genotype emerged about 40 years ago, which is also in accordance to previous data of 39 years for the origin of this genotype [21]. However, it was intriguing that the viruses causing the outbreaks in São Paulo and Rio de Janeiro States during the last three years share a common ancestor few years before the first reported outbreak (median of 9.8 years ago, and lower value of 5.5 years ago), suggesting that this lineage was already circulating in Brazil before its detection in 2007.

In fact, a “silent” introduction of a new DENV into a naïve population has already been documented for DENV4 in Brazil [29], [30]. In 2008, DENV4 was identified in three patients in Manaus, without international travelling history. Based on these data, we can envisage the possibility that the DENV2 found São Paulo and Rio de Janeiro States has maintained a cryptic circulation in such regions or elsewhere (as well as DENV4 in Manaus), until meets favourable conditions to trigger an outbreak. Because the introduction of a new serotype in an endemic area may imply in more severe clinical presentations of dengue disease, our findings shed lights on the importance of a more systematic surveillance of the dengue viruses in endemic regions.

In summary, this work described the molecular and phylogenetic characteristics of DENV2 during the current outbreak in the coastal region of Sao Paulo State, Brazil. The present epidemic in the cities of Guarujá, Santos and surrounding areas, was caused by the same DENV2 lineage that circulated in Rio de Janeiro in 2007 and 2008, but distinct from viruses that circulated during more ancient DENV2 epidemics in Brazil. Also, since the introduction of a new serotype is considered to increase the risk for severe dengue, our data on the silent introduction of DENV2 claims for the risks of cryptic introduction and circulation of new viruses into endemic areas.

This work supports the importance of dengue surveillance improvement and may help developing dengue-control programs. Finally, it is important to remember that the infectious disease dynamic is more than a local problem, involving neighboring regions and the population migration rate. Surveillance programs during non-epidemic seasons and in non-endemic regions may be crucial for early identification of new dengue virus introduction.

## Supporting Information

File S1 A. TMRCA of the DENV2 obtained by Strict and Relaxed Uncorrelated Lognormal Molecular Clock. B. Bayes factor values obtained for molecular clock comparison of DENV2.
(0.03 MB DOC)

Figure S1 Phylogenetic tree of the four DENV serotypes. The maximum clade credibility (MCC) tree was inferred from 93 partial envelope dengue viruses sequences obtained from patients from the cities of Guarujá and Santos (collapsed blue clade) and representative sequences of all DENV serotypes. The posterior probabilities of the key nodes are depicted above the respective node.
(2.95 MB TIF)
