# Supplementary material for: Characterization of Dengue Virus Type 2: New Insights on the 2010 Brazilian Epidemic
Source: PLoS One. 2010 Jul 28;5(7):e11811. doi: 10.1371/journal.pone.0011811 (PMC2911371; doi:10.1371/journal.pone.0011811)
Supplement: File S1 — A. TMRCA of the DENV2 obtained by Strict and Relaxed Uncorrelated Lognormal Molecular Clock. B. Bayes factor values obtained for molecular clock comparison of DENV2. (0.03 MB DOC) [file pone.0011811.s001.doc]

**S1.A.**

|  |  | **TMRCA in years**  **(Upper and Lower)** |  |
| --- | --- | --- | --- |
|  | **SP/RJ lineage** | **American/Asian genotype** | **DENV2** |
| **Relaxed Unc. Log. Molecular Clock** | 9.8 (5.45-16) | 40 (22-68.2) | 123 (58-251.5) |
| **Strict molecular clock** | 7.6 (4.6-10.7) | 26.4 (19-34) | 107 (81.3-134) |

**S1.B**.

| Trace DENV2 | **ln P**  **(molecular clock|data)** | **S.E.** | **Relaxed Clock** | **Strict Clock** |
| --- | --- | --- | --- | --- |
| **Relaxed Clock** | -3163.09 | +/- 0.4 | - | 18.89 |
| **Strict Clock** | -3206.59 | +/- 0.46 | -18.89 | - |
