# Supplementary figures and images for: Characterization of Dengue Virus Type 2: New Insights on the 2010 Brazilian Epidemic
Source: PLoS One. 2010 Jul 28;5(7):e11811. doi: 10.1371/journal.pone.0011811 (PMC2911371; doi:10.1371/journal.pone.0011811)

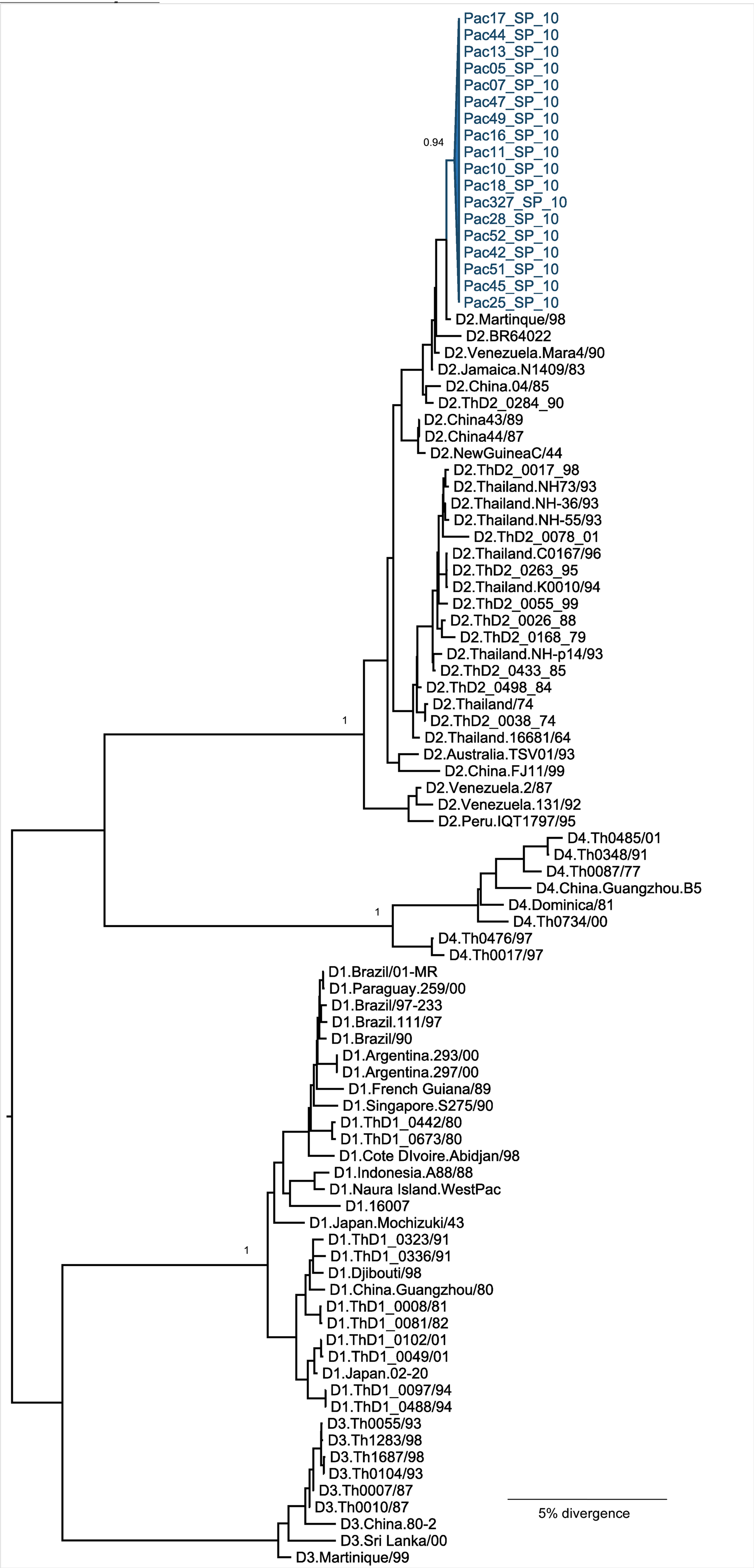

Supplement: Figure S1 — Phylogenetic tree of the four DENV serotypes. The maximum clade credibility (MCC) tree was inferred from 93 partial envelope dengue viruses sequences obtained from patients from the cities of Guarujá and Santos (collapsed blue clade) and representative sequences of all DENV serotypes. The posterior probabilities of the key nodes are depicted above the respective node. (2.95 MB TIF) [file pone.0011811.s002.tif]
